# Supplementary material for: Theta oscillations represent collective dynamics of multineuronal membrane potentials of murine hippocampal pyramidal cells
Source: Commun Biol. 2023 Apr 12;6:398. doi: 10.1038/s42003-023-04719-z (PMC10097823; doi:10.1038/s42003-023-04719-z)
Supplement: Supplementary file 2 — Supplementary Information [file 42003_2023_4719_MOESM2_ESM.pdf]

Supplementary Information for

Theta oscillations represent collective dynamics of multineuronal membrane potentials of murine hippocampal pyramidal cells

Asako Noguchi, Kotaro Yamashiro, Nobuyoshi Matsumoto, Yuji Ikegaya
Correspondence to: asakonoguchi.an@gmail.com

This PDF file includes:

Supplementary Fig. 1 to 13

Supplementary Figure 1: Atropine-sensitive θ_{LFP} in the hippocampus of urethane-anesthetized mice.

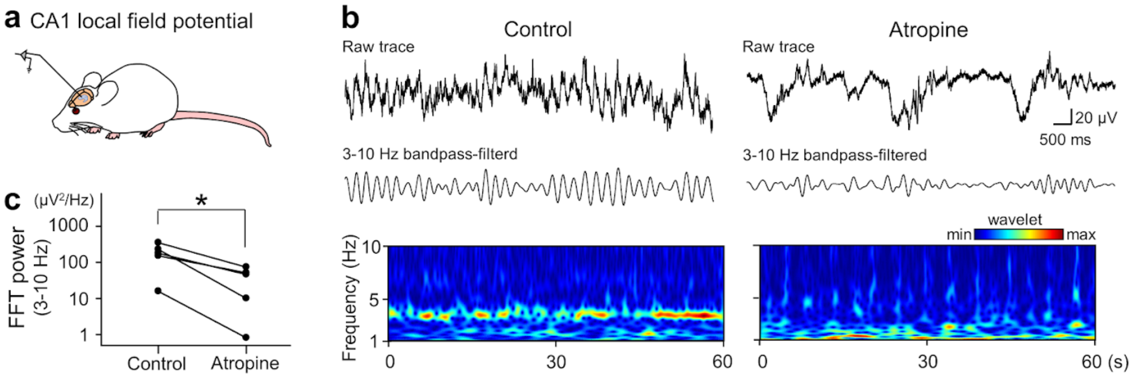

(**a**) Schematic illustration of LFP recordings from the dorsal hippocampal CA1 area.

(**b**) Representative raw traces of CA1 LFPs (top), their 3-10-Hz bandpass-filtered traces (middle), and their wavelet spectrograms (bottom) 10 min before (left, control) and 30 min after intraperitoneal injection of 50 mg/kg atropine (right, atropine).

(**c**) θ_{LFP} power was significantly attenuated after atropine injection. $P = 0.028$, $t_4 = 2.7$, paired t-test, $n = 5$ mice.

Supplementary Figure 2: Highly synchronous θ_{LFP} power changes regardless of the length of the time window.

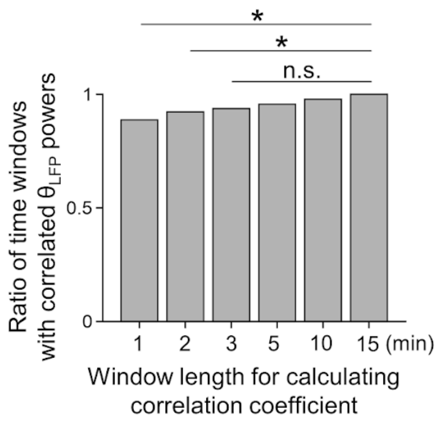

Correlation coefficients of the θ_{LFP} power changes between all 30 pairs of simultaneously recorded LFPs were calculated by using 1-, 2-, 3-, 5-, 10-, and 15-min time windows, and the ratio of the time windows with significant correlations is shown for each window length. The ratio increased as the length of the time window increased, and no significant differences were observed for time windows longer than 3 min (all lengths of time windows, $P = 1.1\times10^{-4}$, $\chi^2 = 25.6$; time windows longer than 2 min, $P = 0.049$, $\chi^2 = 9.6$; time windows longer than 3 min, $P = 0.11$, $\chi^2 = 6.11$, chi-square test, n = 900, 450, 300, 180, 90, and 60 time windows for 1, 2, 3, 5, 10, and 15 min, respectively). For all window lengths, the ratio exceeded 90%, indicating highly synchronous θ_{LFP} power changes regardless of the window length used to calculate the correlation coefficients.

Supplementary Figure 3: Optimization of the threshold for θ_{LFP} detection.

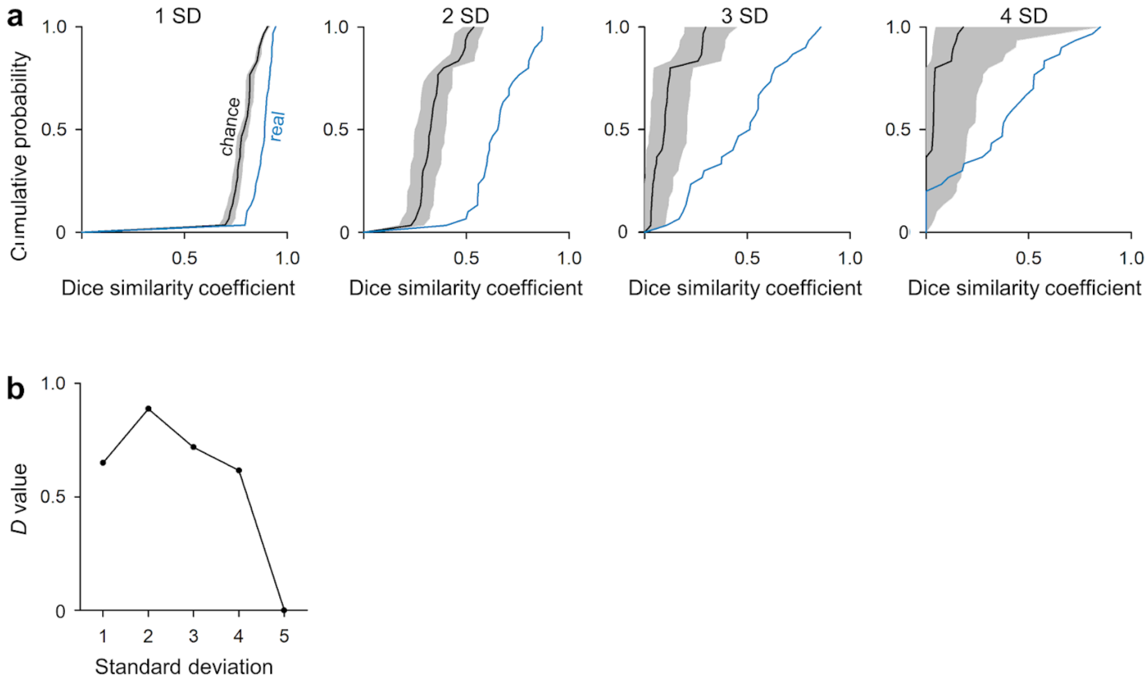

(**a**) Cumulative probabilities of the Dice similarity coefficients between pairs of θ_{LFP}s among four simultaneously recorded CA1 LFPs (see Fig. 1a) at various SDs (1, 2, 3, and 4) for the threshold of oscillation powers above which θ oscillations were identified. The blue lines indicate real data, whereas the black lines and gray shadow areas indicate the mean values and the 95% confidence intervals, respectively, of 10,000 surrogate data in which all detected θ periods were randomly shuffled along the recording time within each recording site.

(**b**) The *D* values of a two-sample Kolmogorov–Smirnov test were calculated from the cumulative distributions in a and plotted against the SDs for the θ thresholds. The *D* values reached a maximum at a threshold of 2 SDs, which was adopted for the detection of θ oscillations in this study.

Supplementary Figure 4: Lack of correlations of θ_{LFP} power with firing rates or peak θ_{Vm} power.

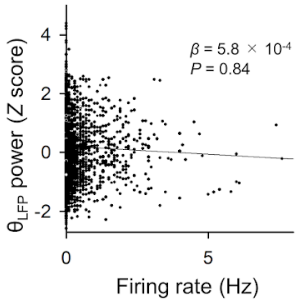

The θ_{LFP} power was not correlated with the firing rates of simultaneously patch-clamped cells. Each dot indicates a single 10-s segment. The black line indicates the line of best fit based on a generalized linear mixed model. $\beta = 5.8 \times 10^{-4}$, $P = 0.84$, $t_{5,387} = 0.20$, $n =$ 5,389 segments from 160 cells.

Supplementary Figure 5: Relationships between θ_{LFP}-θ_{Vm} power correlations and physiological and anatomical properties of individual cells.

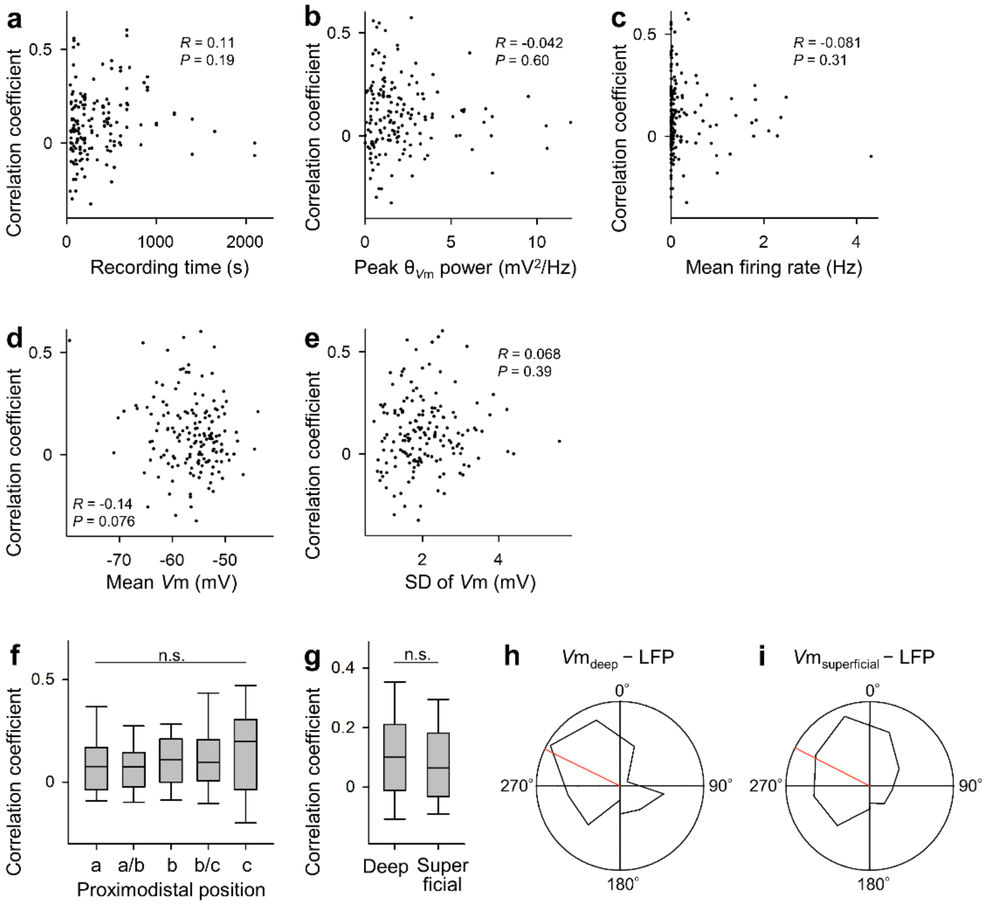

(**a**) Correlation coefficients between θ_{LFP} and θ_{Vm} power changes were plotted against the recording time for each dataset. No significant relationship was found between the correlation coefficients and recording times. $R = 0.11$, $P = 0.19$, *t*-test for correlation coefficients, $n = 160$ cells.

(**b**) No significant relationship was found between the correlation coefficients and the maximal power of θ_{Vm} during the entire recording period. $R = -0.042$, $P = 0.60$, $n = 160$ cells.

(**c**) No significant relationship was found between the correlation coefficients and the mean firing rate of each cell. $R = -0.081$, $P = 0.31$, $n = 160$ cells.

(**d**) No significant relationship was found between the correlation coefficients and the mean *V*m of each cell. $R = -0.14$, $P = 0.076$, $n = 160$ cells.

(**e**) No significant relationship was found between the correlation coefficients and the standard deviation (SD) of *V*m for each cell. $R = 0.068$, $P = 0.39$, $n = 160$ cells.

(**f**) No significant difference was found in the correlation coefficients depending on the cell locations along the proximodistal axis in the CA1 subregion. $P = 0.62$, one-way analysis of variance (ANOVA), n = 27, 41, 45, 11, 11 cells for CA1a, a/b, b, b/c, c, respectively.

(**g**) No significant difference was found in the correlation coefficients depending on the cell locations along the radial axis in the CA1 subregion. $P = 0.27$, $t_{131} = 1.1$, Student's *t*-test, n = 80 and 53 for deep and superficial, respectively.

(**h, i**) Circular distribution of the θ phase difference between LFPs and *V*m when θ_{LFP} and θ_{Vm} occurred simultaneously at similar frequencies (Δ frequency < 0.01 Hz). The datasets were divided into two groups, in which the recorded cells were located in deep (h) or superficial (i) layers. Red lines indicate the mean θ phase differences (-64° and -63° for deep and superficial cells, respectively). No significant difference was found between the preferred phases of deep and superficial cells ($P > 0.1$, $K = 1.8\times10^3$, Kuiper test, n = 151 and 74 periods from 26 and 20 deep and superficial cells, respectively).

Supplementary Figure 6: Weak correlation between θ_{LFP} and θ_{Vm} in awake mice.

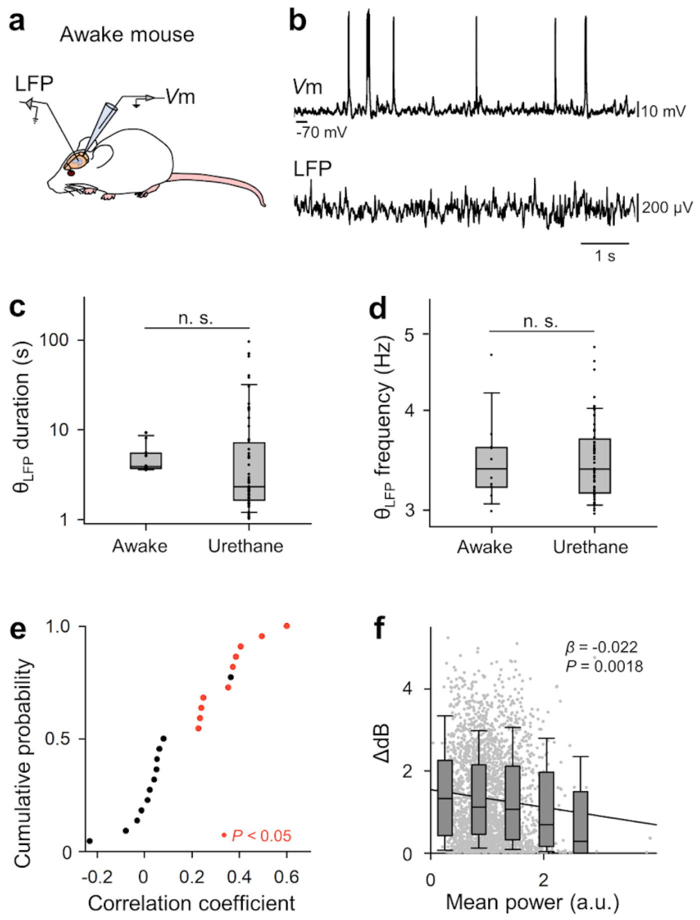

(**a**) Schematic illustration of simultaneous recording of LFPs and *V*m from a CA1 pyramidal cell in a head-fixed unanesthetized mouse.

(**b**) Representative raw traces of simultaneously recorded CA1 LFPs and *V*m of a CA1 pyramidal cell.

(**c**) The mean durations of individual θ_{LFP} periods did not differ between awake and urethane-anesthetized mice. P = 0.40, t_{71} = 0.85, Student's *t*-test, n =10 awake and 63 anesthetized mice.

(**d**) The mean θ_{LFP} frequency did not differ between awake and anesthetized mice. P = 0.96, t_{71} = -0.049, Student's *t*-test, n = 10 awake and 63 anesthetized mice.

(**e**) Cumulative probability distribution of the correlation coefficients between the θ_{LFP} and θ_{Vm} power for all 22 recorded datasets. Red dots indicate cells that showed significant correlations.

(**f**) The difference in frequency between θ_{LFP} and θ_{Vm} for a 1-s segment was negatively correlated with the geometric average of their powers. Each gray dot overlaid on the box plots represents a 1-s segment. The black line indicates the line of best fit based on a generalized linear mixed model. $\beta = -0.022$, $P = 0.0018$, $t_{2,587} = -3.13$, $n = 2{,}589$ θ periods from 22 cells.

Supplementary Figure 7: Weak θ_{Vm} correlation between two CA1 pyramidal cells.

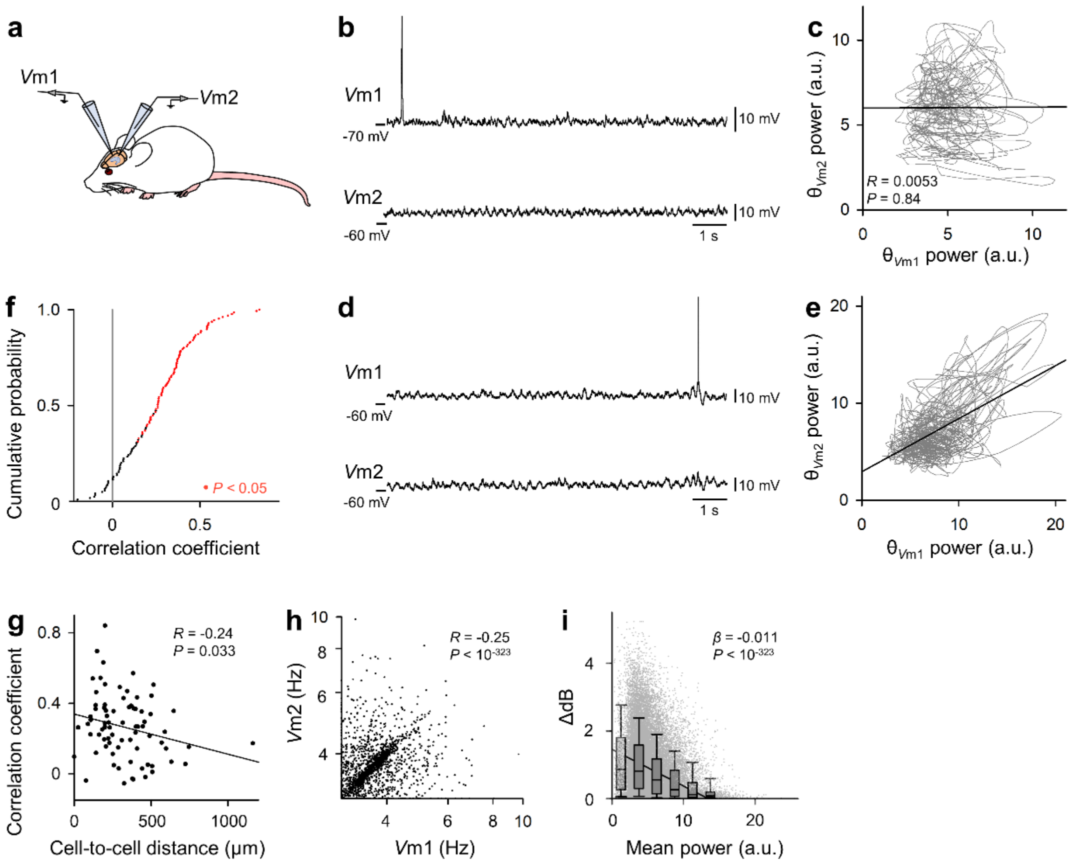

(**a**) Schematic illustration of simultaneous recordings of *V*ms from two CA1 pyramidal cells (*V*m1 and *V*m2).

(**b**) Representative raw traces of *V*ms simultaneously recorded from two CA1 pyramidal cells.

(**c**) Temporal relationship of the θ_{Vm} powers. The θ_{Vm} powers were plotted every 100 ms over the entire recording period for each dataset. No significant correlation was observed (R = 0.0053, P = 0.84, *t*-test for correlation coefficients, n = 1,499 1-s segments). The black line indicates the line of best fit based on least-squares regression.

(**d, e**) Same as b, c, but for a dataset in which a significant positive correlation was observed ($R = 0.56$, $P < 10^{-323}$, n = 2,479 1-s segments).

(**f**) Cumulative probability distribution of the correlation coefficients between the θ_{Vm} powers of all 125 cell pairs. Each red dot indicates a cell pair with a significant correlation.

(**g**) The correlation coefficients of the θ_{Vm} powers (calculated in d) plotted against the

spatial distance between two somata. The black line indicates the line of best fit based on least-squares regression. R = -0.24, P = 0.033, n = 78 cell pairs.

(**h**) Relationships of the $\theta_{V\mathrm{m}}$ frequencies between two cells for periods during which they simultaneously exhibited $\theta_{V\mathrm{m}}$s. Each dot indicates a single co-θ period. R = 0.25, $P < 10^{-323}$, n = 2,027 θ periods from 125 cell pairs.

(**i**) The difference in the $\theta_{V\mathrm{m}}$ frequencies between two pyramidal cells in a given 1-s segment was negatively correlated with the geometric average of the $\theta_{V\mathrm{m}}$ powers. Each gray dot shows a 1-s segment. The black line indicates the line of best fit based on a generalized linear mixed model. β = -0.011, $P < 10^{-323}$, $t_{34,628}$ = -47.3, n = 34,630 θ periods from 125 cell pairs.

Supplementary Figure 8: Similar ratios of cell pairs with correlated θ_{Vm} power changes in the data shown in Fig. 3 and Supplementary Fig. 7.

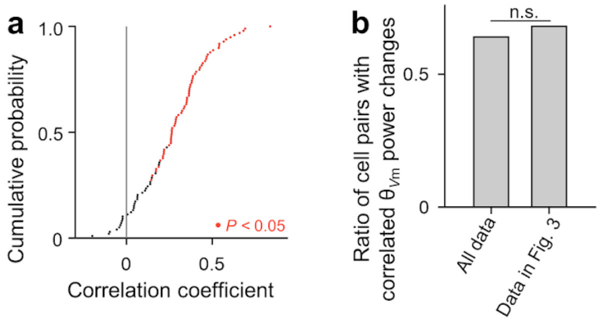

(**a**) Cumulative probability distribution of the correlation coefficients between the θ_{Vm} powers of 98 cell pairs, which were included in the analyses for Fig. 3. Each red dot indicates a cell pair with a significant correlation.

(**b**) Ratios of cell pairs with significantly correlated θ_{Vm} power changes for the data used in the analyses for Supplementary Fig. 7 (left) and only the data used for the analyses in Fig. 3 (right). No significant difference was observed. $P = 0.49$, $\chi^2 = 0.47$, chi-square test, $n = 125$ (all data) and 98 (data in Fig. 3 only) cell pairs.

Supplementary Figure 9: Model selection and parameter tuning for the DNN

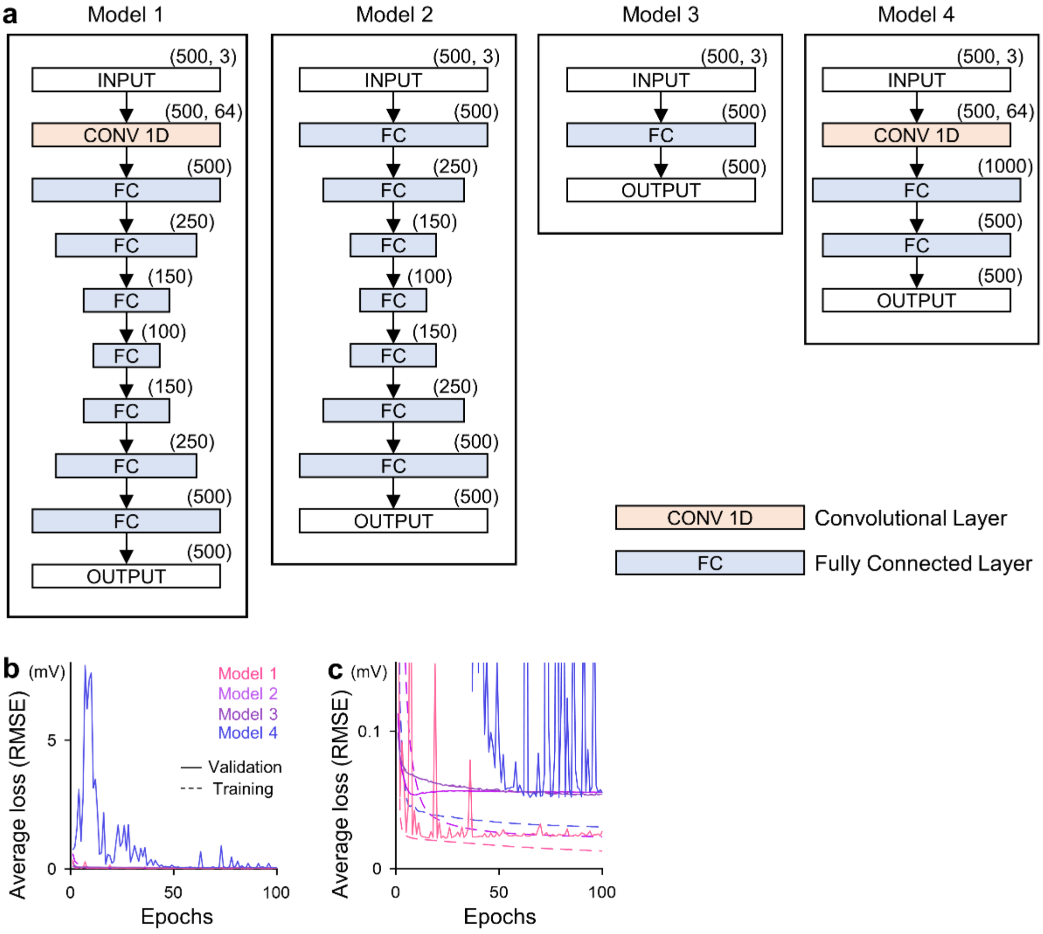

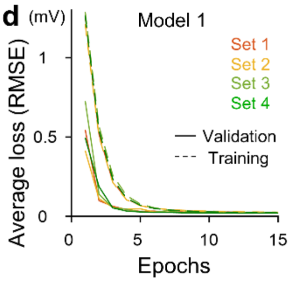

e

	Set 1	Set 2	Set 3	Set 4
Layer 1	250	400	150	250
Layer 2	150	300	100	128
Layer 3	100	200	25	64
Performance (validation RMSE)	0.013	0.089	0.030	0.023

(**a**) Architectures of the four tested neural network models. The numbers indicate the dimensions of each layer. Conv: convolutional, FC: fully connected. The input layer receives three simultaneously recorded *V*ms (*V*m1, *V*m2, and *V*m3) that were bandpass filtered between 3 and 10 Hz (INPUT), and the model was trained to output the corresponding bandpass-filtered LFPs (OUTPUT).

(**b**) Average learning curves across all 5 datasets for the four model architectures, indicated by different colors. Loss was computed as the root mean squared error (RMSE). Dotted and solid lines show training and validation loss, respectively.

(**c**) Same as left but with an enlarged vertical axis.

(**d**) Average learning curves across all 5 datasets for the four sets of parameters used in the Model 1 architecture, indicated by different colors. Dotted and solid lines show training and validation loss, respectively.

(**e**) Four sets of parameters and the validation RMSE for each set. Set 1 was selected because these parameters obtained the smallest validation loss.

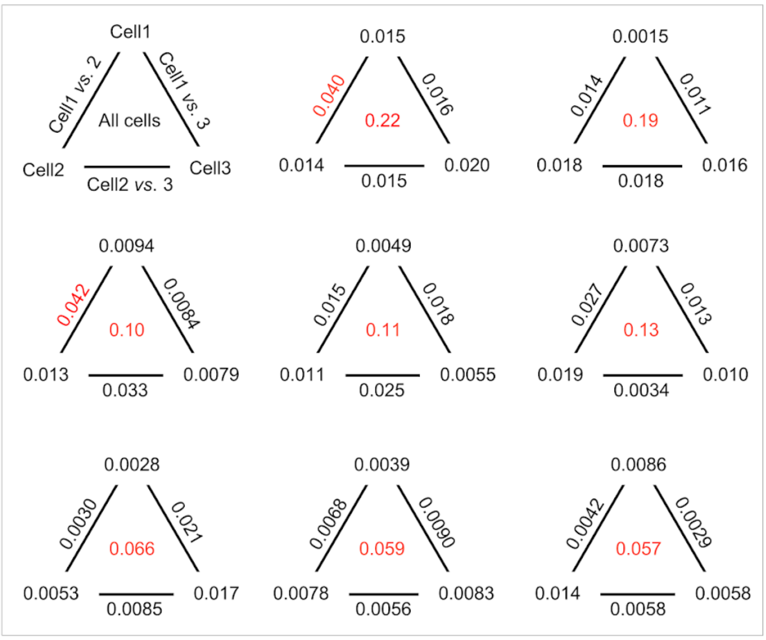

Supplementary Figure 10: *D* values of RMSEs for θ_{LFP} prediction based on 1, 2, and 3 θ_{Vm}s between real data and shuffled data.

The same data as in Fig. 5d but represented as triangles for all 8 datasets of simultaneous recordings. The *D* values for the predictions from 3, 2 and 1 *V*ms are located at the center, edges, and corners of each triangle, respectively, as exemplified in the upper left panel. Red values indicate significant *D* values ($P < 0.05$, two-sample Kolmogorov–Smirnov test).

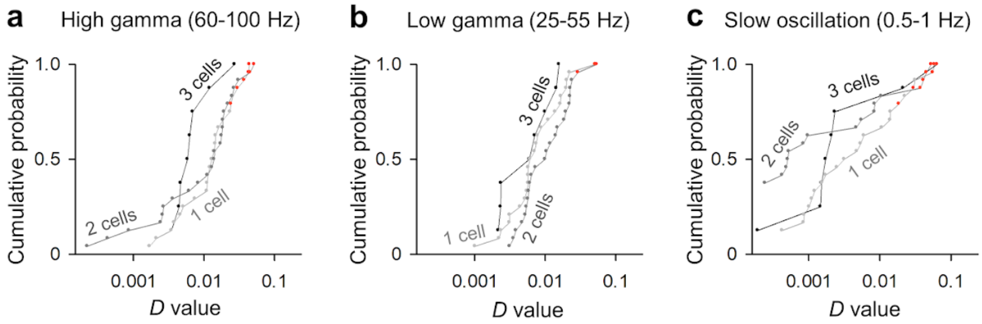

Supplementary Figure 11: LFP prediction from *V*ms in frequency bands other than θ.

(**a**) The same plot as Fig. 5d but for the high-gamma frequency band (60-100 Hz). Red dots indicate datasets with significant *D* values. No significant differences were observed depending on the number of cells used for the prediction. $D_{1\ vs.\ 2\ \mathrm{cells}} = 0.17$, $P_{1\ vs.\ 2\ \mathrm{cells}} = 0.86$, $D_{1\ vs.\ 3\ \mathrm{cells}} = 0.50$, $P_{1\ vs.\ 3\ \mathrm{cells}} = 0.066$, $D_{2\ vs.\ 3\ \mathrm{cells}} = 0.46$, $P_{2\ vs.\ 3\ \mathrm{cells}} = 0.11$, two-sample Kolmogorov–Smirnov test. $n = 24$, 24, and 8 datasets for 1, 2, and 3 cells, respectively.

(**b**) The same as a but for the low-gamma frequency band (25-55 Hz). No significant differences were observed depending on the number of cells used for the prediction. $D_{1\ vs.\ 2\ \mathrm{cells}} = 0.21$, $P_{1\ vs.\ 2\ \mathrm{cells}} = 0.62$, $D_{1\ vs.\ 3\ \mathrm{cells}} = 0.25$, $P_{1\ vs.\ 3\ \mathrm{cells}} = 0.79$, $D_{2\ vs.\ 3\ \mathrm{cells}} = 0.78$, $P_{2\ vs.\ 3\ \mathrm{cells}} = 0.29$.

(**c**) The same as a but for slow oscillations (0.5-1 Hz). Slow oscillations were better predicted from 1 *V*m than 2 *V*ms. $D_{1\ vs.\ 2\ \mathrm{cells}} = 0.46$, $P_{1\ vs.\ 2\ \mathrm{cells}} = 0.0082$, $D_{1\ vs.\ 3\ \mathrm{cells}} = 0.33$, $P_{1\ vs.\ 3\ \mathrm{cells}} = 0.43$, $D_{2\ vs.\ 3\ \mathrm{cells}} = 0.50$, $P_{2\ vs.\ 3\ \mathrm{cells}} = 0.066$.

Supplementary Figure 12: Weak power but similar temporal relationships among LFPs and *V*ms in frequency bands other than θ.

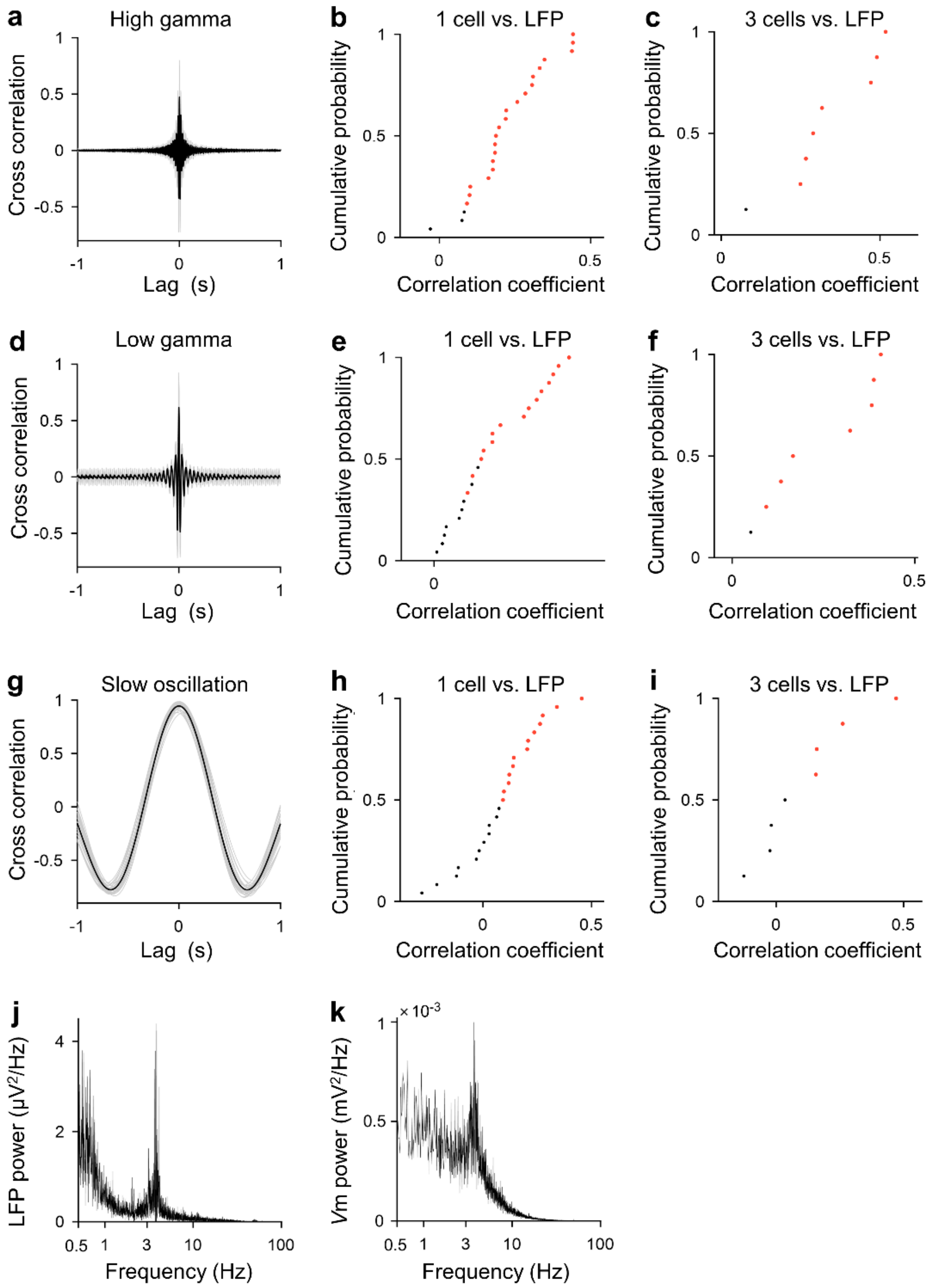

(**a-c**) Correlated high gamma powers among LFPs and *V*ms. (**a**) Cross-correlograms of the 30 pairs of simultaneously recorded LFPs bandpass filtered at 60-100 Hz (gray line) and their mean (black). (**b**) Cumulative probability distribution of the correlation coefficients between θ_{LFP} and θ_{Vm} powers of all 24 cells used in the DNN analysis in Fig.

5. (**c**) Cumulative probability distribution of the correlation coefficients between θ_{LFP} powers and the mean θ_{Vm} powers of three simultaneously recorded cells for all 8 datasets used in the DNN analysis. Each red dot indicates a dataset with a significant positive correlation.

(**d-f**) Same as a-c, but for the low gamma frequency band (25-55 Hz).

(**g-i**) Same as a-c, but for the slow oscillations (0.5-1 Hz).

(**j**) LFP power spectrum averaged across all 8 datasets used in the DNN analysis. The most dominant peak was observed between 3 and 10 Hz.

(**k**) Same as d, but for *V*m. Dominant peaks were observed between 3 and 10 Hz.

Supplementary Figure 13: Correlations of θ traces and powers between LFPs and *V*ms.

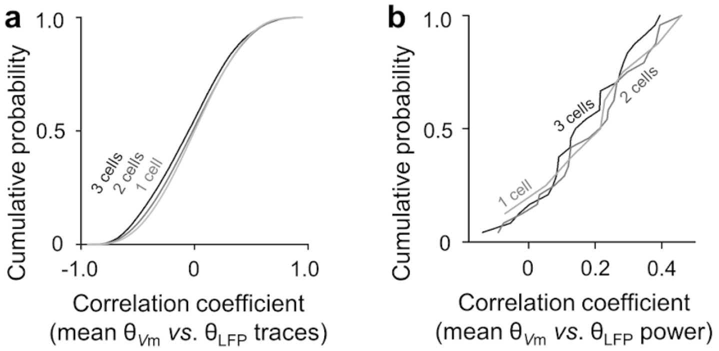

(**a**) Cumulative probability distributions of the correlation coefficients between θ_{LFP} traces and the mean θ_{Vm} traces of 1, 2, or 3 cells simultaneously recorded. Darker colors indicate the results for a larger number of cells. The correlation coefficients decreased as the number of cells increased, indicating that the mean θ_{Vm} traces of more cells were less similar to the θ_{LFP} trace. $D_{1\ vs.\ 2\ cells} = 0.029$, $P_{1\ vs.\ 2\ cells} = 6.5\times10^{-42}$, $D_{1\ vs.\ 3\ cells} = 0.049$, $P_{1\ vs.\ 3\ cells} = 4.1\times10^{-59}$, $D_{2\ vs.\ 3\ cells} = 0.021$, $P_{2\ vs.\ 3\ cells} = 1.3\times10^{-12}$, two-sample Kolmogorov–Smirnov test, n = 112,740, 112,740, and 37,580 1-s segments from 8 mice for 1, 2, and 3 cells, respectively.

(**b**) Cumulative probability distributions of the correlation coefficients between θ_{LFP} powers and the mean θ_{Vm} powers of 1, 2, or 3 cells simultaneously recorded. No significant differences were observed depending on the number of cells. $D_{1\ vs.\ 2\ cells} = 0.17$, $P_{1\ vs.\ 2\ cells} = 0.86$, $D_{1\ vs.\ 3\ cells} = 0.25$, $P_{1\ vs.\ 3\ cells} = 0.79$, $D_{2\ vs.\ 3\ cells} = 0.17$, $P_{2\ vs.\ 3\ cells} = 0.99$.
