# Supplementary material for: Theta oscillations represent collective dynamics of multineuronal membrane potentials of murine hippocampal pyramidal cells
Source: Commun Biol. 2023 Apr 12;6:398. doi: 10.1038/s42003-023-04719-z (PMC10097823; doi:10.1038/s42003-023-04719-z)
Supplement: Supplementary file 3 — Description of Additional Supplementary Files [file 42003_2023_4719_MOESM3_ESM.pdf]

Description of Additional Supplementary Files

File name: Supplementary Data

Description: The source data behind the graphs in the paper
