# Supplementary material for: Theta oscillations represent collective dynamics of multineuronal membrane potentials of murine hippocampal pyramidal cells
Source: Commun Biol. 2023 Apr 12;6:398. doi: 10.1038/s42003-023-04719-z (PMC10097823; doi:10.1038/s42003-023-04719-z)
Supplement: Supplementary file 5 — Reporting Summary [file 42003_2023_4719_MOESM5_ESM.pdf]

Corresponding author(s): DBPR COMMSBIO-22-3193A

Last updated by author(s): Feb 28, 2023

Reporting Summary

Nature Portfolio wishes to improve the reproducibility of the work that we publish. This form provides structure for consistency and transparency in reporting. For further information on Nature Portfolio policies, see our Editorial Policies and the Editorial Policy Checklist.

Statistics

For all statistical analyses, confirm that the following items are present in the figure legend, table legend, main text, or Methods section.

n/a	Confirmed	
☐	☒	The exact sample size (*n*) for each experimental group/condition, given as a discrete number and unit of measurement
☐	☒	A statement on whether measurements were taken from distinct samples or whether the same sample was measured repeatedly
☐	☒	The statistical test(s) used AND whether they are one- or two-sided<br>*Only common tests should be described solely by name; describe more complex techniques in the Methods section.*
☒	☐	A description of all covariates tested
☐	☒	A description of any assumptions or corrections, such as tests of normality and adjustment for multiple comparisons
☐	☒	A full description of the statistical parameters including central tendency (e.g. means) or other basic estimates (e.g. regression coefficient) AND variation (e.g. standard deviation) or associated estimates of uncertainty (e.g. confidence intervals)
☐	☒	For null hypothesis testing, the test statistic (e.g. *F*, *t*, *r*) with confidence intervals, effect sizes, degrees of freedom and *P* value noted<br>*Give P values as exact values whenever suitable.*
☒	☐	For Bayesian analysis, information on the choice of priors and Markov chain Monte Carlo settings
☒	☐	For hierarchical and complex designs, identification of the appropriate level for tests and full reporting of outcomes
☐	☒	Estimates of effect sizes (e.g. Cohen's *d*, Pearson's *r*), indicating how they were calculated

Our web collection on statistics for biologists contains articles on many of the points above.

Software and code

Policy information about availability of computer code

Data collection	All electrophysiological data was acquired with pClamp 10.3 (Molecular Devices), an open source software.
Data analysis	All offline computational analyses except Figure 5 and Supplementary Figure 9 were performed using MATLAB R2017b. The DNN based analyses in Figure 5 and Supplementary Figure 9 were performed using Python 3.9.1. Each analysis procedure is described in necessary detail in the Method section for others to execute. Any analysis code and analyzed data are available by sending a request to the corresponding author.

For manuscripts utilizing custom algorithms or software that are central to the research but not yet described in published literature, software must be made available to editors and reviewers. We strongly encourage code deposition in a community repository (e.g. GitHub). See the Nature Portfolio guidelines for submitting code & software for further information.

Data

Policy information about availability of data

All manuscripts must include a data availability statement. This statement should provide the following information, where applicable:

- Accession codes, unique identifiers, or web links for publicly available datasets
- A description of any restrictions on data availability
- For clinical datasets or third party data, please ensure that the statement adheres to our policy

The data that support the findings of the study are available from the corresponding author upon reasonable request.

Human research participants

Policy information about studies involving human research participants and Sex and Gender in Research.

Reporting on sex and gender	N/A
Population characteristics	N/A
Recruitment	N/A
Ethics oversight	N/A

Note that full information on the approval of the study protocol must also be provided in the manuscript.

Field-specific reporting

Please select the one below that is the best fit for your research. If you are not sure, read the appropriate sections before making your selection.

☒ Life sciences ☐ Behavioural & social sciences ☐ Ecological, evolutionary & environmental sciences

For a reference copy of the document with all sections, see nature.com/documents/nr-reporting-summary-flat.pdf

Life sciences study design

All studies must disclose on these points even when the disclosure is negative.

Sample size	Sample size (the number of triple patching datasets and single whole-cell recordings in awake condition) was determined by previous publications that performed in vivo multiple whole-cell recordings from the hippocampus (Jouhanneau et al., 2018, 2019). Single and dual whole-cell recordings in anesthetized condition were obtained more frequently in this order in the process of achieving triple whole-cell recordings, which resulted in larger number of data than triple patching datasets. No statistical methods were used to predetermine the sample size.
Data exclusions	For all analyses of membrane potentials, we only used the data in which the mean resting potential were below -50 mV and the action potentials were above -20 mV. For the analyses defining theta periods, the data without theta periods in the analyzed signals were excluded. For the analyses calculating the anatomical distances between LFP electrodes and patch-clamped cells, we only used the data in which we could identified both of their anatomical locations. For the analyses using the DNN, only the datasets with recording durations of longer than 3 minutes were used for enough number of data points to train the DNN. All threshold and criteria used for data analyses are clearly described in the Methods section.
Replication	All conclusions of this study are based on recordings of populations of cells.
Randomization	For Supplementary Figure 1, mice were randomly assigned to atropine and control groups. For Supplementary Figure 6, mice were randomly assigned to two recording groups in awake and anesthetized conditions. For the other analyses, no randomization was performed.
Blinding	No blinding was done in this study because knowledge of experimental conditions was required during data collection.

Reporting for specific materials, systems and methods

We require information from authors about some types of materials, experimental systems and methods used in many studies. Here, indicate whether each material, system or method listed is relevant to your study. If you are not sure if a list item applies to your research, read the appropriate section before selecting a response.

Materials & experimental systems

n/a	Involved in the study	
☒	☐	Antibodies
☒	☐	Eukaryotic cell lines
☒	☐	Palaeontology and archaeology
☐	☒	Animals and other organisms
☒	☐	Clinical data
☒	☐	Dual use research of concern

Methods

n/a	Involved in the study	
☒	☐	ChIP-seq
☒	☐	Flow cytometry
☒	☐	MRI-based neuroimaging

Animals and other research organisms

Policy information about studies involving animals; ARRIVE guidelines recommended for reporting animal research, and Sex and Gender in Research

Laboratory animals	In this study, we used postnatal day 28- to 45-day-old male ICR mice (Japan SLC, Shizuoka, Japan).
Wild animals	The study did not involve wild animals.
Reporting on sex	This study cannot address if the findings apply to both sexes or not because only male mice were used.
Field-collected samples	The study did not involve samples collected from the field.
Ethics oversight	nimal experiments were performed with the approval of the animal experiment ethics committee at the University of Tokyo (approval numbers: P29-9) and in accordance with the University of Tokyo guidelines for the care and use of laboratory animals. These experimental protocols were conducted in accordance with the Fundamental Guidelines for the Proper Conduct of Animal Experiments and Related Activities in Academic Research Institutions (Ministry of Education, Culture, Sports, Science and Technology, Notice No. 71 of 2006), the Standards for Breeding and Housing of and Pain Alleviation for Experimental Animals (Ministry of the Environment, Notice No. 88 of 2006) and the Guidelines on the Method of Animal Disposal (Prime Minister's Office, Notice No. 40 of 1995).

Note that full information on the approval of the study protocol must also be provided in the manuscript.
